# Historical heritage–MultiCriteria Decision Method (H-MCDM) to prioritize intervention strategies for the adaptive reuse of valuable architectural assets

**DOI:** 10.1016/j.mex.2023.102487

**Published:** 2023-12-01

**Authors:** Gabriella Maselli, Pasquale Cucco, Antonio Nesticò, Federica Ribera

**Affiliations:** Department of Civil Engineering, University of Salerno, Fisciano, Italy

**Keywords:** Historical heritage, Adaptive reuse, MultiCriteria Decision Methods, Analytic hierarchy process, Evaluation indicators, Economic analysis, H-MCDM (Historical heritage – MultiCriteria Decision Methods)

## Abstract

Identifying the most suitable strategy for the adaptive reuse of historic architectural heritage is a complex challenge. This is because each rehabilitation project of an abandoned historic building must not only ensure the simultaneous pursuit of social, cultural and financial goals, but also the transmission of tangible and intangible values to future generations.

In this context, the use of multi-criteria approaches helps to synthesise stakeholders' preferences based on multiple and conflicting criteria, with the aim of promoting sustainable solutions for the restoration and valorisation of valuable architectural assets. To this end, we characterise the Historical heritage - MultiCriteria Decision Method (H-MCDM), an analysis and evaluation tool aimed at identifying the best adaptive reuse of historic buildings considering the principles of architectural conservation and both economic and socio-cultural values. It is a model based on the Analytic Hierarchy Process (AHP), which employs 7 evaluation indicators, chosen after a critical and interpretative analysis of both the sustainability principles promoted by the United Nations and UNESCO, and the ICOMOS quality principles.

The H-MCDM is structured on the following five logical-operational steps: (i) identification of strategies for adaptive reuse of the historic building; (ii) construction of the matrices of the pairwise comparisons of the criteria and estimation of the vector of the weights of the criteria; (iii) construction of the matrices of the pairwise comparisons of the alternatives against each evaluation criterion and estimation of the local scores of the alternatives; (iv) consistency checks of the decision matrices; (v) calculation of the total priority and ranking of the HBU strategies for historic assets.

The application to a case study demonstrates that, due to its adaptability and versatility, the H-MCDM can be a valid and consistent decision support in public resource allocation processes for the rehabilitation of architectural heritage.

• The H-MCDM is a flexible and practical tool to evaluate the best strategy for adaptive reuse of historical heritage.
• The H-MCDM is an AHP-based model which employs 7 indicators relating to the social, cultural and economic spheres.
• The evaluation indicators are chosen in accordance with 2030 SDGs and European Quality Principles.

## Graphical abstract

**Figure:**
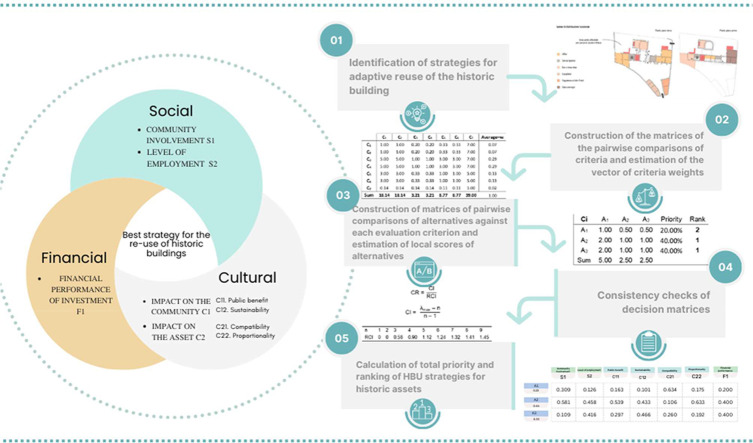

Specifications table

**Table:** 

Subject area	Engineering
More specific subject area	*Decision Making Science. Cultural heritage conservation. Economic evaluation of projects.*
Name of your method	*H-MCDM (Historical heritage – MultiCriteria Decision Methods)*
Name and reference of original method	*Analytic Hierarchy Process and Highest and Best Use*
Resource availability	*N.A.*

## Background

Cultural Heritage (CH) can play a strategic role in the pursuit of sustainable development [1]. This especially in territorial contexts such as Europe where a significant share of the architectural capital is made up of historic sites and buildings, not infrequently abandoned or undervalued [2,3]. The crucial role of CH is increasingly recognised by major national and global institutions, as demonstrated by the United Nations 2030 Agenda for Sustainable Development, an action plan that sets 17 Sustainable Development Goals (SDGs) and 169 interdependent targets to address the three dimensions of sustainability. Specifically, SDG 11.4 emphasises the need to strengthen global efforts to protect and safeguard cultural and natural heritage, thereby recognising the intrinsic importance of protecting and enhancing architectural, cultural, and environmental assets [4]. In 2015, UNESCO promoted a concrete policy to integrate a sustainable development perspective into the World Heritage (WH) Convention processes. The aim is to strengthen the centrality of the role of historical and architectural heritage in social, environmental and economic challenges, thus promoting peace and security in line with the UN development agenda (2015). Again, International Council on Monuments and Sites (ICOMOS) in its recent essay of 2020 “European Quality Principles for EU-funded Interventions with Potential Impact upon Cultural Heritage” [5] emphasises the need to share quality principles in the conservation and sustainable management of cultural heritage. This to ensure the quality of any intervention and avoid erasing valuable traces of the past due to insensitive modernisation and urbanisation [6], [7], [8].

The role of cultural heritage within the circular economy and circular cities is also receiving increasing attention [9], [10], [11]. Strategies to conserve and reuse resources to extend their life cycle help achieve circular economy goals, close or slow down resource cycles, reduce resource consumption, and prevent waste production [1,12]. Furthermore, according to Labadi et al. [13], non-renewable heritage resources must be conserved to contribute to sustainable development and growth. A now widely recognised strategy for preserving cultural heritage is that of ‘adaptive reuse’, by which is meant the process of extending the useful life of heritage by providing it with a (new) use and thus preserving it [14,15]. In this regard, Glumac and Islam [16] argue that adapting instead of demolishing, whenever possible, “is an essential ingredient in changing the construction industry towards a more sustainable future and conserving valuable resources for time to come”. However, identifying sustainable and architecturally permissible reuse actions is a complex challenge. Firstly, because the Adaptive Reuse of Cultural Heritage (ARCH) must simultaneously redevelop historic buildings, prolonging their life through multiple uses, always respecting the principles of integrity and authenticity of cultural heritage [17]. In addition, since it is a multidisciplinary process, in which architecture, archaeology, geography, art history, urban planning, engineering, economics, environmental science, sociology and political action must necessarily work together for the public good, there are multiple actors involved in the protection and enhancement of CH [3]. As Plevoets and van Cleempoel [18] put it, the ARCH is an interdisciplinary and multi-stakeholder process that fosters the environmental, social, cultural and economic dimensions of sustainable development. For instance, from an environmental perspective, it can prevent/reduce the production of demolition waste [19] or enable the conservation of embodied energy [15]. Furthermore, according to Sesana et al. [20], threats to heritage related to climate change, e.g. accelerating degradation processes, can be addressed within the ARCH framework by reducing the vulnerability of heritage to climate change and climate change impacts. From a socio-cultural perspective, ARCH can help maintain heritage attributes and values [21,22] and generate employment [23]. Finally, ARCH strategies can generate higher profits and sometimes contain maintenance and management costs [19,21,24,25]. According to Tsoukiàs et al. [26], the decision-making problems in question refer to collective decisions that are characterised by the following five main complexities: (i) use of public resources, (ii) presence of multiple stakeholders, (iii) long-time horizons, (iv) need for legitimation and accountability and (v) need for deliberation.

For these reasons, it becomes essential to define new approaches and tools capable of addressing the challenges of the ARCH and implementing the principles set out in international and national recommendations. This is with the aim of preserving historical assets and revitalising them in a circular economy perspective. Specifically, we intend to propose an innovative multi-criteria approach to select the best new use to be attributed to abandoned historic buildings that still retain values to be protected and passed on to future generations. Specifically, we define a Historical heritage - MultiCriteria Decision Method (H-MCDM), in which the principles of architectural conservation are merged with economic and socio-cultural values in a methodological ‘unicum’, in which the choice of evaluation indicators is derived from a critical and interpretative analysis of the principles of sustainability promoted by the United Nations and UNESCO, and of the ICOMOS principles of quality. It is an Analytic Hierarchy Process (AHP)-based model that, by employing a set of 7 easy-to-estimate indicators, can be widely used by planners and policymakers whenever it is necessary to identify the best strategy for adaptive reuse of historic buildings.

## Method approach

Following the overview presented in the previous section, we define a new model to assess the best adaptive reuse for historic buildings based on AHP. several decision support tools have been developed over time in the context of choosing the best ARCH strategy. To name just a few, Ferretti et al. [27] define a multi-criteria decision-making model (MCDM) based on multi-attribute value theory (MAVT) to decide the best use and select the best function for historic buildings. The analytical hierarchy process (AHP), proposed by Saaty [28], has also been extensively tested in the context of architectural heritage reuse [8,29,30]. In fact, it is a method that can be used to choose, classify, prioritise, and put a more equitable allocation of resources. classification, prioritisation, resource allocation. Wang and Zeng [31] use the Analytic Network Process (ANP) and the Delphi Method to identify the most effective reuse alternative for historical sites in Taiwan. Bottero et al. [32] focus on the topic of adaptive reuse of heritage by proposing a new application of the preference ranking organisation method for evaluation enrichment (PROMETHEE). To address the complexity of the decision-making problem regarding the choice about the highest and best adaptive reuse of cultural heritage, Della Spina [33] defines an approach based on the combination of three multi-criteria methods: the MacBeth method, the Analytic Hierarchy Process (AHP), and the Evaluation of Mixed data (EVAMIX). Again, Dell'Ovo et al. [34] use the Novel Approach to Imprecise Assessment and Decision Environments (NAIADE) to define the most suitable function for the adaptive reuse of the Castello Visconteo in Cusago (Italy).

Although the literature review revealed the use of different MCDMs in decision-making processes aimed at enhancing the architectural heritage, we considered the AHP as the most fitting method to select the best reuse strategy for historic buildings. Firstly, because AHP allows the result to be expressed through a list of priorities, where each alternative is given a score [28,35,36]. In contrast, compromise ranking methods (VIKOR, Vise Kriterijumska Optimizacija I Kompromisno Resenje) [37] only score alternatives if specific acceptability criteria are met: thus, they do not return a priority list but only the acceptable alternatives. Outranking methods such as ELimination Et Choix Traduisant la REalité (ELECTRE), on the other hand, only inform on the winning alternatives and are only performant with a limited number of criteria and alternatives [38,39].

A second critical issue common to different AHPs is the assignment of weights to each criterion, which is done by comparing the different evaluation criteria in pairs. In the case of the AHP, the definition of the weights to be assigned to the criteria is more flexible, since it is possible to assign different weights to criteria that are on different hierarchical levels. In the case of other methods, such as ELECTRE, VIKOR, and TOPSIS (Technique for Order Preference by Similarity to Ideal Solution) all criteria are compared in pairs with each other, identifying them as belonging to a single level [40,41].

A further aspect to highlight concerns the choice of criteria used to define multi-criteria schemes: these are too often qualitative indicators and most often chosen according to the peculiarities of the case study. The H-MCDM, on the other hand, employs a set of seven criteria expressed mainly through quantitative indicators and selected while considering: the financial sustainability of the investment project; the principles of sustainable development; the guidelines of international bodies for the protection of historic buildings and sites. Therefore, these are criteria with general validity that relate to three different sustainability pillars: social (S), cultural (C), and financial (F). These first level criteria (S, C and F) allow a coherent and comprehensive assessment of the performance resulting from the reuse of the historic building.

### Social sustainability

The social aspect or criterion is assessed through the sub-criteria: community involvement (S_1_) and level of employment (S_2_). These two criteria are expressed through indicators that consider the Quality Principles and the 2030 SDGs, which are respectively:

– average daily number of users (indicator S_1_) and
– number of new employees for each functional proposal (indicator S_2_).

To estimate indicator S_1_, it is necessary to preliminarily evaluate for each functional alternative the appropriate dimensions to be allocated to each room in the building and consequently assess the maximum number of users to be hosted, on the basis of the minimum requirements provided for by the regulations and the comfort parameters, also starting from socio-economic analyses carried out in the context in which the building is placed.

To assess indicator S_2_, it is necessary to foresee the activities to be carried out for each functional alternative and therefore estimate, also based on available space, the number of new occupants.

### Cultural sustainability

The cultural dimension of sustainability is a function of:

– impact on the community (C_1_) and
– impact on the asset (C_2_).

The sub-criterion ‘impact on the community’ (C_1_) synthesises the potential impacts that the different re-functionalisation alternatives could have on the local community, both in terms of the benefits that each specific function could bring from a socio-cultural point of view, and in terms of the perception engendered in the community with respect to the new feature of the historic building. The new function must therefore represent the community's needs and interests, encouraging the reintroduction of the disused or undervalued historic building into a renewed circuit of life.

The ‘impact on the community’ (C_1_) is in turn expressed as a function of public benefit (C_11_) and sustainability (C_12_).

There are three aspects that make it possible to define public benefit (C_11_): cultural impact on the community, satisfaction of local needs and achievement of the SDG Goals. In other words, C_11_ represents the set of objectives that the functional destination can pursue, bringing to the local community a benefit and interest that satisfy current and future needs. The indicator should be assessed by taking into account which and how many SDG goals the function was able to achieve. In addition, the interaction that the function to create with the community due to the public use of the asset should be assessed.

Sustainability (C_12_) is defined by three factors: utility of the function, enhancement of the building and management of the building. C_12_ indicates the ability of the function to last over time and to be subject to sustainable management that ensures the best cyclical maintenance of the asset. This indicator is assessed above all with a view to whether the proposed functions can enhance the value of the building and also be considered suitable by the community to which it belongs, which is thus able through new use to perceive the spirit and memory of the work still alive.

The sub-criterion ‘impact on assets’ (C_2_) is intended to assess the material impact of the proposed functional uses in terms of quantity and quality of interventions. The latter must be assessed in the context of their level of invasiveness, considering how much and how they are grafted into the historic material, and tend to be limited to a minimum. In this context, the potential reversibility of such interventions is fundamental to ensure greater flexibility in acting on the recovered asset, eliminating additions if necessary and re-evaluating design choices that are no longer effective. The proposed functions are also evaluated in relation to how they can be installed while respecting the original layout of the structure, to minimise design distortions that risk distorting the building's main historical characteristics.

C_2_ in turn includes two third-level criteria: compatibility (C_21_) and proportionality (C_22_). C_21_ indicates the degree of appropriateness of the function with respect to the historical-architectural characteristics of the property. This criterion expresses the ability of the projects to pursue the criterion of minimum intervention, minimum invasiveness, and potential reversibility of the planned interventions. C_22_ indicates the extent to which the function of use is pertinent to the original characteristics of the structure, to its planimetric conformation, to the arrangement of the rooms and connectives, and the extent to which it succeeds in enhancing its spatial and architectural peculiarities without distorting the layout.

The aspects that characterise the third level criteria are developed from the recommendations contained in the ICOMOS document. They attempt to answer the initial questions posed by the international institution for each individual Quality Principle, accompanied by considerations made in accordance with the universally recognised principles that guarantee compatible interventions in the historic built environment.

All four indicators are rated on a qualitative scale of preferences with values between 1 and 9.

### Financial sustainability

The financial criterion (F) aims to summarise the financial performance of the project (F_1_).

F_1_ is expressed through the return on investment (ROI), which is understood as the rate of return on a company's investment. The ROI of each alternative is approximated to the average ROI of the sector in which the investment falls. Since these are ex-ante financial evaluations, the indicator can be estimated on the balance sheet data of similar companies. These data are provided by databases such as Bureau van Dijk's ORBIS, which provide information on companies geographically distributed around the world.

**Fig. 1:**
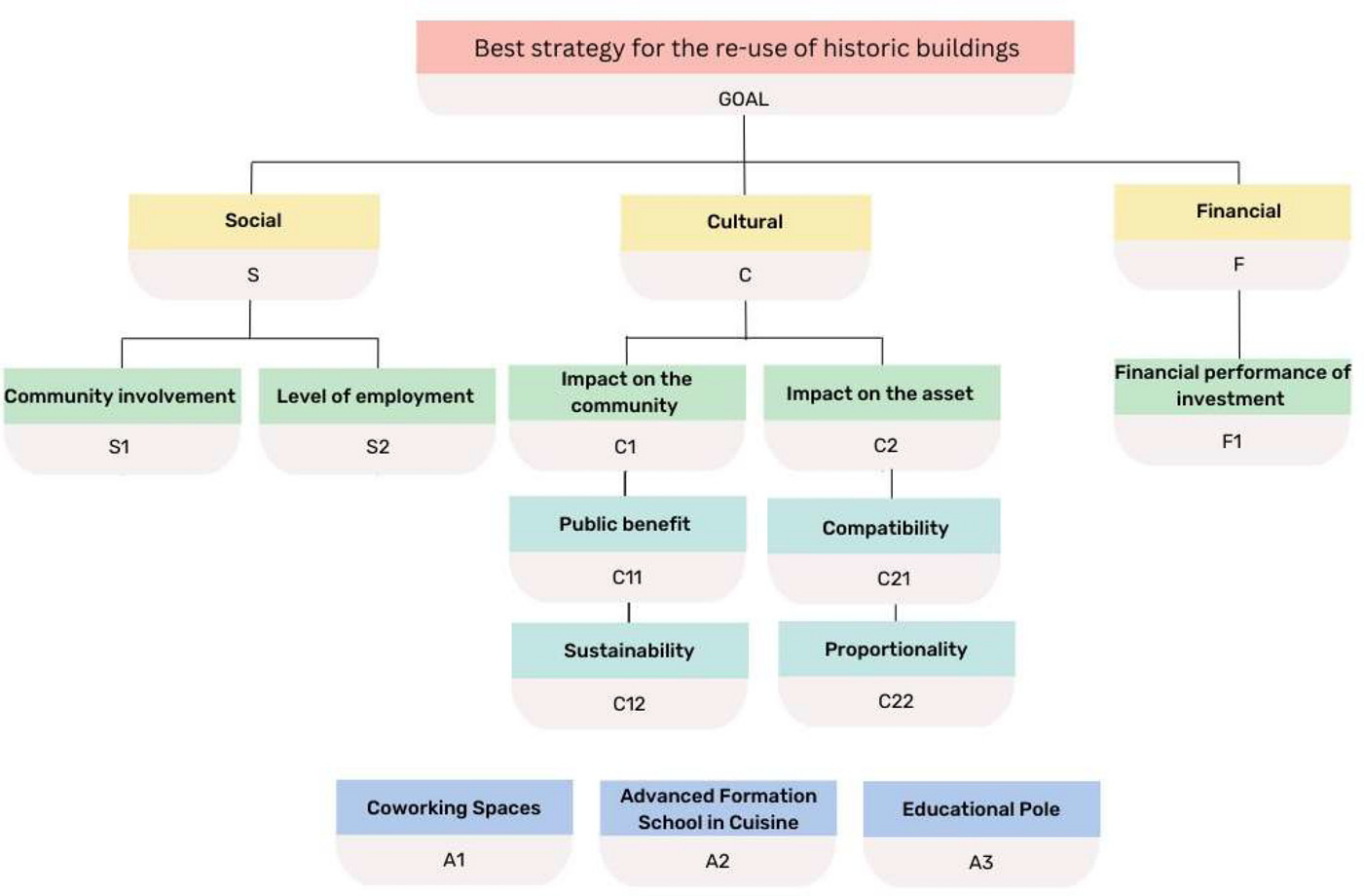
Hierarchical structure of the problem.

Fig. 1 shows the hierarchical structure of the decision-making problem.

## H-MCDM structure

The H-MCDM is structured on the following five logical-operational steps:

(i) Identification of strategies for adaptive reuse of the historic building;
(ii) Construction of the matrices of the pairwise comparisons of the criteria and estimation of the vector of the weights of the criteria
(iii) Construction of the matrices of the pairwise comparisons of the alternatives against each evaluation criterion and estimation of the local scores of the alternatives;
(iv) Consistency checks of the decision matrices;
(v) Calculation of total priority and ranking of HBU strategies for historic assets.

**Fig. 2:**
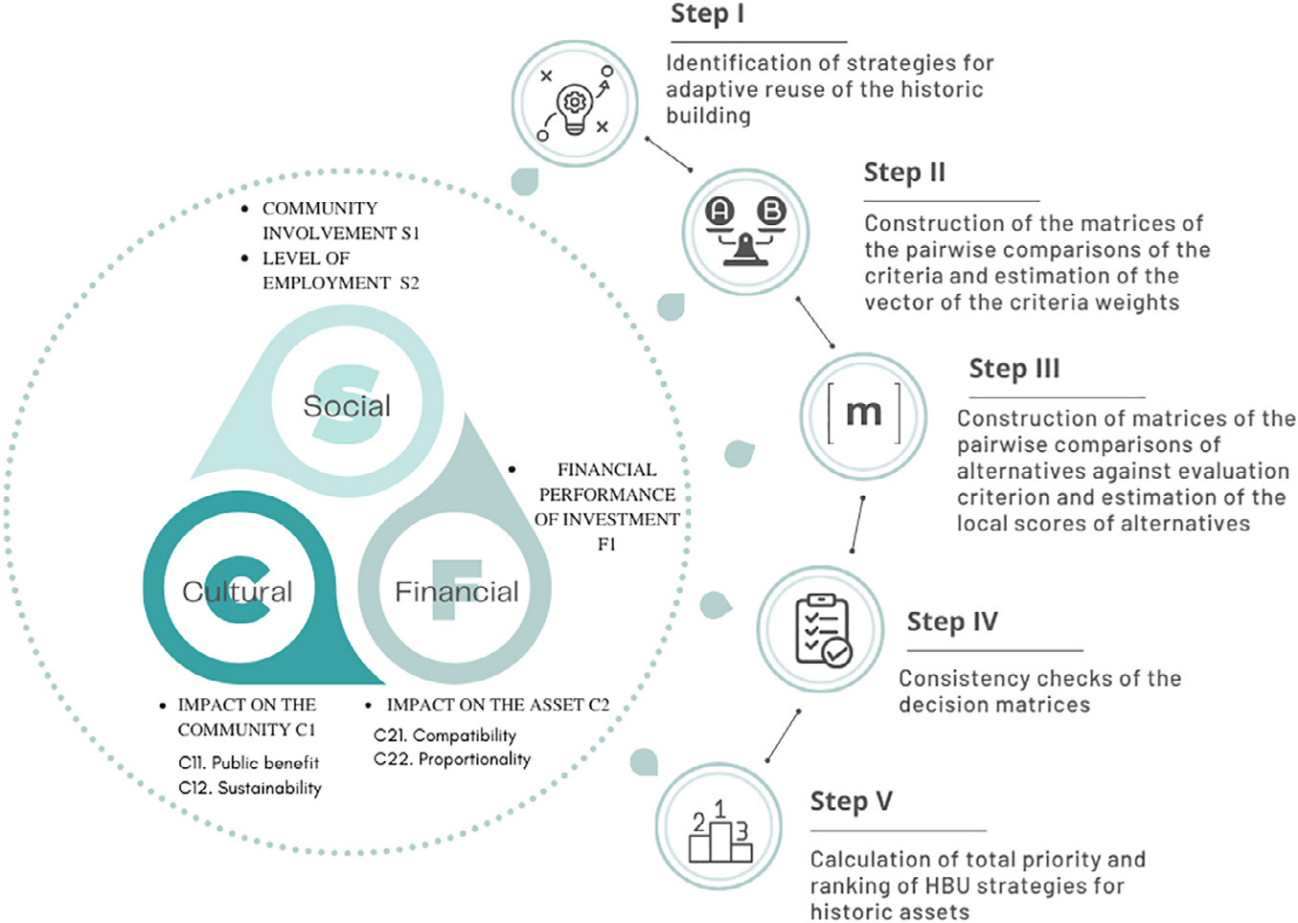
H-MCDM structure.

Fig. 2 summarises the H-MCDM structure.

The H-MCDM is applied to identify the best strategy for the historical re-use of an 18th century villa in the province of Naples (Campania Region, Italy).

### Step i. Identification of strategies for adaptive reuse of the historic building

The choice of possible uses to be attributed to the historic building in ruins and subject to restoration is a critical preliminary step. First, public and private operators must select alternative functions after a careful social, cultural and economic analysis of the context in which the building is located, and after analysing the needs and requirements of communities, local associations and stakeholders. In addition, the design of reuse strategies for ruined historic buildings pose challenges: (a) *structural*, as microshocks and environmental shocks can lead to new and more dangerous functional mechanisms over time, as well as generate infiltration; (b) *aesthetic*, as the old-new balance must always be guaranteed; (c) *functional*, as the change of use must be compatible with the original layout; (d) *technological*, as the location of these works often does not favour wide and convenient accessibility. Finally, functional uses must be: technically feasible, urbanistically admissible, economically viable, and architecturally compatible.

The three alternatives, chosen based on statistical surveys on the number and qualification of inhabitants, the functional needs expressed in municipal and regional programmes and inter-municipal collaboration and growth strategies, are as follows:

A_1_. Co-working space,
A_2_. Advanced formation school in cuisine,
A_3_. Educational pole.

*Step ii. Construction of the matrices of the pairwise comparisons of criteria and estimation of the vector of criteria weights*

To establish the importance of each criterion, all criteria belonging to the same hierarchical level are compared in pairs. The result of the comparison is called the dominance coefficient a_ij_ and indicates the greater or lesser importance of the first element i in relation to the second element j. Therefore, if w_i_ is the weight of the first element and w_j_ is the weight of the second element, then a_ij_ = w_i_/w_j_.

Pairwise comparisons are attributed using Saaty's semantic scale, which relates the first nine integers with as many judgements expressing the possible results of the comparison (Table 1).

**Table 1:** Saaty's semantic scale.

*Intensity*	1	3	5	7	9	2, 4, 6, 8
*Linguistic*	Equal	Moderate	Strong	Demonstrated	Extreme	Intermediate values

Usually, the ratio w_i_/w_j_ is unknown, so it is necessary to find that value of αij such that α_ij_ ≈ w_i_/w_j_. W denotes the matrix of weights and w the column vector of the variables sought. W is positive, invertible, square (n x n) with n number of elements to be compared [35,36]. Therefore, we have:

(1) $$W\;\cdot \;w\;=\;\left[\begin{array}{cccc} \frac{w_{1}}{w_{1}} & \frac{w_{1}}{w_{2}} & \ldots & \frac{w_{1}}{w_{n}}\\ \frac{w_{2}}{w_{1}} & \frac{w_{2}}{w_{2}} & \ldots & \frac{w_{2}}{w_{n}}\\ \vdots & \vdots & \ddots & \vdots \\ \frac{w_{n}}{w_{1}} & \frac{w_{n}}{w_{2}} & \ldots & \frac{w_{n}}{w_{n}} \end{array}\right]\cdot \left[\begin{array}{c} w_{1}\\ w_{2}\\ \vdots \\ w_{n} \end{array}\right]=\left[\begin{array}{c} n\;w_{1}\\ n\;w_{2}\\ \vdots \\ n\;w_{n} \end{array}\right]=n\;\left[\begin{array}{c} w_{1}\\ w_{2}\\ \vdots \\ w_{n} \end{array}\right]=n\;\cdot \;w$$

In contracted form:

(2) W·w=n·w

The matrix W has as its corresponding eigenvalue the vector of the variables sought. If only an estimate of the ratios w_i_/w_j_ (i, j = 1, 2, ..., n) is available, the eigenvalue method can still be used. The values found will be closer to the exact values the more consistent (or consistent) the estimates provided for the w_i_/w_j_ ratios are with each other [42]. Therefore, the maximum eigenvalue λmax is close to the value n, which can be used as an estimate of w.

(3) $$W\;\cdot \;n\;=\;\lambda_{\text{max}}\cdot \;w\;\;\text{with}\;\;\lambda_{\text{max}}\;\geq \;n$$

As an alternative to the ‘rigorous’ calculation of the eigenvalue and eigenvector, the vector w can be determined more rapidly by the ‘approximate’ method described below: (i) evaluating the normalised matrix W_N_ by dividing each element of the matrix W by the sum of the dominance coefficients present in the i-th column; (ii) evaluating the vector of priorities or criteria weights w(c), assuming that its i-th element is equal to the geometric mean of the elements present on the i-th row of W_N_ [43,44].

With reference to the case study, the weights of the evaluation criteria are defined with reference to the following scenarios:

(a) criteria of the same level all have the same weight;
(b) the weights of the criteria are different and are assigned by a panel of experts;
(c) the criteria belong to one level and have equal weight;
(d) the criteria belong to a single level and have different weights.

Table 2a, Table 2b, Table 2c, Table 2d shows the pairwise comparisons matrix, the normalised matrix and the vector of criteria weights for the four scenarios.

**Table 2a:** Estimation of Criteria Weights (scenario a).

	S	C	F	Normalized matrix			Average = w_i_
**S**	1.00	1.00	1.00	0.333	0.333	0.333	0.333
**C**	1.00	1.00	1.00	0.333	0.333	0.333	0.333
**F**	1.00	1.00	1.00	0.333	0.333	0.333	0.333
**Sum**	3.00	3.00	3.00				

	C_1_	C_2_	Normalized matrix		Average = w_i_		

**C_1_**	1.00	1.00	0.5	0.5	0.5		
**C_2_**	1.00	1.00	0.5	0.5	0.5		
**Sum**	2.00	2.00

**Table 2b:** Estimation of Criteria Weights (scenario b).

	S	C	F	Normalized matrix			Average = w_i_
**S**	1.00	0.33	5.00	0.24	0.22	0.38	0.28
**C**	3.00	1.00	7.00	0.71	0.68	0.54	0.64
**F**	0.20	0.14	1.00	0.05	0.10	0.08	0.07
**Sum**	4.20	1.47	13.00				

	C_1_	C_2_	Normalized matrix		Average = w_i_		

**C_1_**	1.00	1.00	0.5	0.5	0.5		
**C_2_**	1.00	1.00	0.5	0.5	0.5		
**Sum**	2.00	2.00

**Table 2c:** Estimation of Criteria Weights (scenario c).

	C_1_	C_2_	C_3_	C_4_	C_5_	C_6_	C_7_	Normalized matrix							Average = w_i_
**C_1_**	1.00	1.00	1.00	1.00	1.00	1.00	1.00	0.14	0.14	0.14	0.14	0.14	0.14	0.14	0.14
**C_2_**	1.00	1.00	1.00	1.00	1.00	1.00	1.00	0.14	0.14	0.14	0.14	0.14	0.14	0.14	0.14
**C_3_**	1.00	1.00	1.00	1.00	1.00	1.00	1.00	0.14	0.14	0.14	0.14	0.14	0.14	0.14	0.14
**C_4_**	1.00	1.00	1.00	1.00	1.00	1.00	1.00	0.14	0.14	0.14	0.14	0.14	0.14	0.14	0.14
**C_5_**	1.00	1.00	1.00	1.00	1.00	1.00	1.00	0.14	0.14	0.14	0.14	0.14	0.14	0.14	0.14
**C_6_**	1.00	1.00	1.00	1.00	1.00	1.00	1.00	0.14	0.14	0.14	0.14	0.14	0.14	0.14	0.14
**C_7_**	1.00	1.00	1.00	1.00	1.00	1.00	1.00	0.14	0.14	0.14	0.14	0.14	0.14	0.14	0.14
**Sum**	**7.00**	**7.00**	**7.00**	**7.00**	**7.00**	**7.00**	**7.00**

**Table 2d:** Estimation of Criteria Weights (scenario d).

	C_1_	C_2_	C_3_	C_4_	C_5_	C_6_	C_7_	Normalized matrix							Average = w_i_
**C_1_**	1.00	1.00	0.20	0.20	0.33	0.33	7.00	0.06	0.06	0.06	0.06	0.04	0.04	0.18	0.07
**C_2_**	1.00	1.00	0.20	0.20	0.33	0.33	7.00	0.06	0.06	0.06	0.06	0.04	0.04	0.18	0.07
**C_3_**	5.00	5.00	1.00	1.00	3.00	3.00	7.00	0.28	0.28	0.31	0.31	0.34	0.34	0.18	0.29
**C_4_**	5.00	5.00	1.00	1.00	3.00	3.00	7.00	0.28	0.28	0.31	0.31	0.34	0.34	0.18	0.29
**C_5_**	3.00	3.00	0.33	0.33	1.00	1.00	5.00	0.17	0.17	0.10	0.10	0.11	0.11	0.13	0.13
**C_6_**	3.00	3.00	0.33	0.33	1.00	1.00	5.00	0.17	0.17	0.10	0.10	0.11	0.11	0.13	0.13
**C_7_**	0.14	0.14	0.14	0.14	0.11	0.11	1.00	0.01	0.01	0.04	0.04	0.01	0.01	0.03	0.02
**Sum**	**18.14**	**18.14**	**3.21**	**3.21**	**8.77**	**8.77**	**39.00**								1.00

*Step iii. Construction of matrices of pairwise comparisons of alternatives against each evaluation criterion and estimation of local scores of alternatives*

To establish the score of each alternative with reference to each criterion, we construct a matrix A_Ci_ for each evaluation criterion, in which the alternatives under consideration are compared in pairs. . A_Ci_ is positive, invertible, and square, of the type m x m, where m is the number of alternatives to be compared against the i-th sub-criterion C_i_.

The mathematical steps for estimating the local scores of each alternative for each criterion s_Ci(Ai)_ are the same as those analysed in Section 2. Thus, we first construct the scalar decision matrix, which reports the value of each evaluation indicator for the m alternatives (Table 3).

**Table 3:** Scalarized decision matrix.

	Co-working Space (A_1_)	Advanced Formation School in Cuisine (A_2_)	Educational Pole (A_3_)
Average daily number of users	137	177	73
Number of new employs	18	30	27
Public Benefit	5	7	6
Sustainability	3	6	7
Compatibility	7	3	5
Proportionality	4	7	5
ROI	8.87%	9.71%	10.00%

Finally, the matrices of pairwise comparisons (as many as there are evaluation criteria), the corresponding normalised matrix and the vector of local scores are defined (Table 4).

**Table 4:** Comparison matrices in pairs between the alternatives with respect to each criterion.

**Average daily number of users**	A_1_	A_2_	A_3_	Normalized matrix			w, Priority	Rank
A_1_	1.00	0.50	3.00	0.300	0.294	0.333	30.93%	**2**
A_2_	2.00	1.00	5.00	0.601	0.588	0.556	58.15%	**1**
A_3_	0.33	0.20	1.00	0.099	0.118	0.111	10.93%	**3**
Sum	3.33	1.70	9.00					

**Number of new employs**	A_1_	A_2_	A_3_	Normalized matrix			w, Priority	Rank

A_1_	1.00	0.25	0.33	0.125	0.111	0.142	12.59%	**2**
A_2_	4.00	1.00	1.00	0.500	0.444	0.429	45.79%	**1**
A_3_	3.00	1.00	1.00	0.375	0.444	0.429	41.62%	**1**
Sum	8.00	2.25	2.33					

**Public Benefit**	A_1_	A_2_	A_3_	Normalized matrix			w, Priority	Rank

A_1_	1.00	0.33	0.50	0.167	0.180	0.143	16.33%	**3**
A_2_	3.00	1.00	2.00	0.500	0.546	0.571	53.93%	**1**
A_3_	2.00	0.50	1.00	0.333	0.273	0.286	29.74%	**2**
Sum	6.00	1.83	3.50					

**Sustainability**	A_1_	A_2_	A_3_	Normalized matrix			w, Priority	Rank

A_1_	1.00	0.25	0.20	0.100	0.111	0.091	10.07%	**3**
A_2_	4.00	1.00	1.00	0.400	0.444	0.455	43.30%	**2**
A_3_	5.00	1.00	1.00	0.500	0.444	0.455	46.63%	**1**
Sum	10.00	2.25	2.20					

**Compatibility**	A_1_	A_2_	A_3_	Normalized matrix			w, Priority	Rank

A_1_	1.00	5.00	3.00	0.654	0.556	0.693	63.40%	**1**
A_2_	0.20	1.00	0.33	0.131	0.111	0.076	10.60%	**3**
A_3_	0.33	3.00	1.00	0.216	0.333	0.231	26.00%	**2**
Sum	1.53	9.00	4.33					

**Proportionality**	A_1_	A_2_	A_3_	Normalized matrix			w, Priority	Rank

A_1_	1.00	0.25	1.00	0.167	0.158	0.200	17.50%	**3**
A_2_	4.00	1.00	3.00	0.667	0.633	0.600	63.32%	**1**
A_3_	1.00	0.33	1.00	0.167	0.209	0.200	19.18%	**2**
Sum	6.00	1.58	5.00					

**ROI**	A_1_	A_2_	A_3_	Normalized matrix			w, Priority	Rank

A_1_	1.00	0.50	0.50	0.200	0.200	0.200	20.00%	**2**
A_2_	2.00	1.00	1.00	0.400	0.400	0.400	40.00%	**1**
A_3_	2.00	1.00	1.00	0.400	0.400	0.400	40.00%	**1**
Sum	5.00	2.50	2.50					

*Step iv. Consistency checks of decision matrices*

For measuring consistency, however, it is necessary to have the value of λ_max_, which can be calculated by multiplying the row vector “*Sum*” by the column vector “w, *Priority*”, determined by the ‘approximate’ method described above.

Alternatively, theory suggests estimating the vector “w, *Priority”*, by normalising the matrix W through the ratio of each of its elements to the sum of the elements of the same column and then calculating the arithmetic mean of each of its rows [45].

Specifically, once the weights have been assigned to the criteria and the scores to the alternatives, it is necessary to verify the mutual consistency of the assigned weights, estimating the Consistency Ratio (CR):

(4) $$\text{CR}\;=\;\frac{\text{CI}}{\text{RCI}}$$

In (4), the consistency index (CI) measures the consistency of pairwise comparisons:

(5) $$\text{CI}\;=\;\frac{\lambda_{\text{max}}\;-\;n}{n\;-\;1}$$

In (5) n represents the number of variables to be compared.

The Random Consistency Index (RCI) is a function of the number n of variables (Table 5). Each RCI value is obtained as a random average consistency index based on a sample of 500 randomly generated binary comparison matrices with CI less than 10%.

**Table 5:** Valori di RCI.

N	1	2	3	4	5	6	7	8	9
RCI	0	0	0.58	0.90	1.12	1.24	1.32	1.41	1.45

Finally, the CR value allows the consistency of binary comparisons to be checked. These comparisons are adequately consistent if: CR < 5%, for n = 3; CR < 9%, for n = 4; CR < 10%, for n > 4. With reference to the case study, Table 6 returns the consistency checks for each matrix of pairwise comparisons.

**Table 6:** Consistency checks.

Criteria weight consistency checks (scenario 4, step ii)						
	λ_max_	n	CI	RCI	CR	Check
	7.5185824	7	0.08643	1.32	0.0655	Yes
Consistency checks scores (step iii)						

	λ_max_	n	CI	RCI	CR	Check

Average daily number of users	3.0005532	3	0.000277	0.58	0.0005	Yes
Number of new employs	3.0073023	3	0.003651	0.58	0.0063	Yes
Public Benefit	3.0075917	3	0.003796	0.58	0.0065	Yes
Sustainability	3.0069024	3	0.003451	0.58	0.006	Yes
Compatibility	3.0498946	3	0.024947	0.58	0.043	Yes
Proportionality	3.0094459	3	0.004723	0.58	0.0081	Yes
ROI	3.0000000	3	0.000000	0.58	0.00%	Yes

### Step v. Calculation of total priority and ranking of HBU strategies for historic assets

This step consists of summarising the judgements by tracing the hierarchy upwards. In other words, knowing both the score of the m alternatives, for each sub-category or higher-order category, and the weight of each criterion, a weighted sum is made. That is, the products of the local scores s_Ci(Ai)_ that each alternative has in relation to each criterion and the weight of the relevant criterion w_(Ci)_, are added together, thus obtaining the total score, W_TOT(Ai)_, of the m alternatives:

$$W_{\text{TOT}(\text{Ai})}=s_{C1(\text{Ai})}\cdot w_{\left(C1\right)}+s_{C2(\text{Ai})}\cdot w_{\left(C2\right)}+\ldots +s_{\text{Cn}(\text{Ai})}\cdot w_{\left(\text{Cn}\right)}$$

The analysis thus carried out leads to the identification of the best alternative, which is the one with the highest total score.

**Fig. 3:**
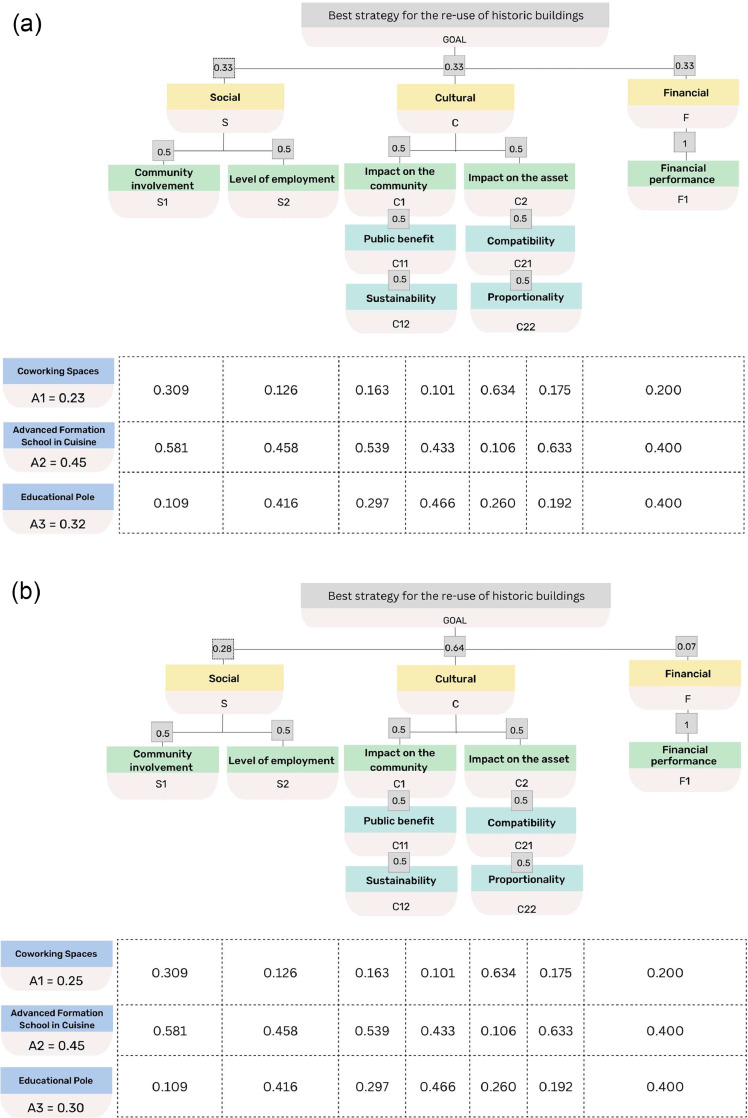

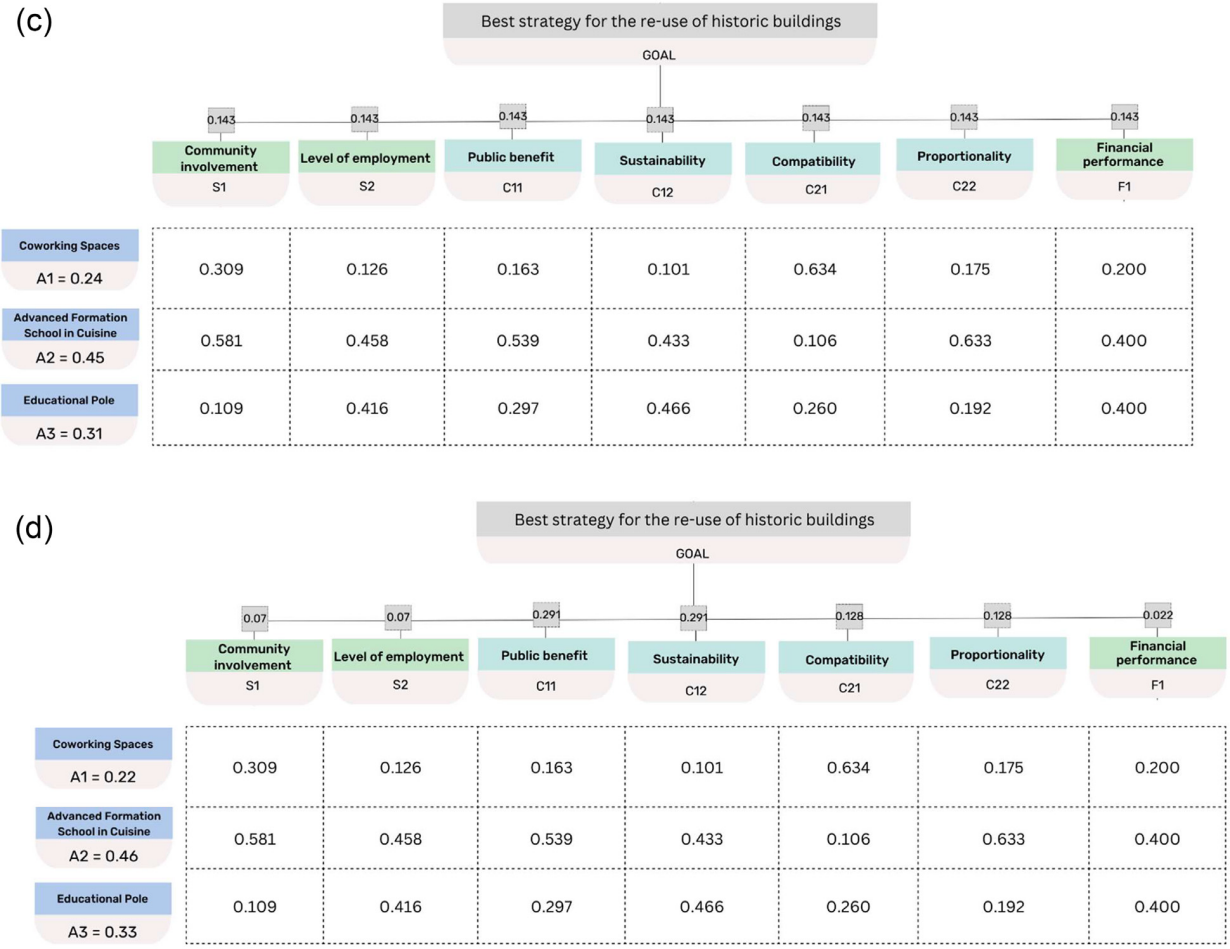
(a) Priority matrix and alternatives ranking (scenario a). (b) Priority matrix and alternatives ranking (scenario b). (c) Priority matrix and alternatives ranking (scenario c). (d) Priority matrix and alternatives ranking (scenario d).

The total priority was assessed for the three alternative strategies A_1_, A_2_ and A_3_, with reference to the four scenarios. The elaborations show that in all analysed scenarios the winning alternative is the advanced formation school in cuisine (A_2_), followed by the educational centre (A_3_) and finally coworking spaces (A_1_). Moreover, the total score that each alternative obtained also varies little from one scenario to the next, demonstrating in fact that the four scenarios are almost equivalent. Fig. 3 gives the results of the elaborations.

## Conclusions

The H-MCDM represents a simple, practical, and flexible approach that can be employed by decision-makers and analysts whenever the best adaptive re-use strategy for historic and architectural heritage needs to be chosen. H-MCDM is based on AHP, which is among the most flexible multi-criteria approaches. Firstly, because it allows the result to be expressed through a list of priorities, where each alternative is assigned a score. Furthermore, through the AHP it is possible to assign different weights to criteria that are on different levels of the hierarchy. Furthermore, the method is based on a set of seven criteria, rigorously chosen considering both the SDG 2030 and the ICOMOS essay ‘European Quality Principles for EU-Funded Interventions with Potential Impact on Cultural Heritage’. Therefore, these are generally valid criteria, to which the analysts may decide to add others if required by the specific case study.

Additional advantages of the method include: the use of readily available data from project results, databases or secondary sources; simple calculation procedures, thus stimulating a wider and more widespread use of the H-MCDM, even by public administrations themselves.

H-MCDM was successfully applied for the selection of the Highest and Best Use of an 18^th^ century villa in the province of Naples (Campania region, Italy). However, due to its adaptability and versatility, H-MCDM can be applied to a wide range of case studies, constituting a valid and scientific decision support with important policy implications in the processes of allocation of public funding to historical heritage.

## CRediT authorship contribution statement

**Gabriella Maselli:** Writing – original draft, Methodology, Data curation, Visualization. **Pasquale Cucco:** Writing – review & editing, Data curation, Visualization. **Antonio Nesticò:** Conceptualization, Methodology, Supervision, Writing – review & editing, Visualization. **Federica Ribera:** Conceptualization, Supervision, Visualization, Writing – review & editing.

## Declaration of Competing Interest

The authors declare that they have no known competing financial interests or personal relationships that could have appeared to influence the work reported in this paper.

## Data Availability

No data was used for the research described in the article.
